# Acet­oxy-γ-valerolactone

**DOI:** 10.1107/S1600536813013561

**Published:** 2013-05-25

**Authors:** Cameron Tristram, Graeme J. Gainsford, Simon Hinkley

**Affiliations:** a Carbohydrate Chemistry Group, Callaghan Innovation Research Limited, PO Box 31-310, Lower Hutt, New Zealand

## Abstract

Levulinyl cellulose esters have been produced as an effective renewable binder for architectural coatings. The title compound, C_7_H_10_O_4_ (systematic name: 2-methyl-5-oxo­tetra­hydro­furan-2-yl acetate), assigned as the esterifying species, was isolated and crystallized to confirm the structure. In the crystal, the mol­ecules pack in layers parallel to (102) utilizing weak C—H⋯O inter­actions.

## Related literature

For related structures, see: Cai *et al.* (2004 ▶). For hydrogen-bonding motifs, see: Bernstein *et al.* (1995 ▶). For background information, see: Bredt (1886 ▶); Rasmussen & Brattain (1949 ▶); Suami & Day (1959 ▶); Glenny *et al.* (2012 ▶). For a previous description of the title compound but without supporting crystal structure data, see: Bell & Covington (1975 ▶).
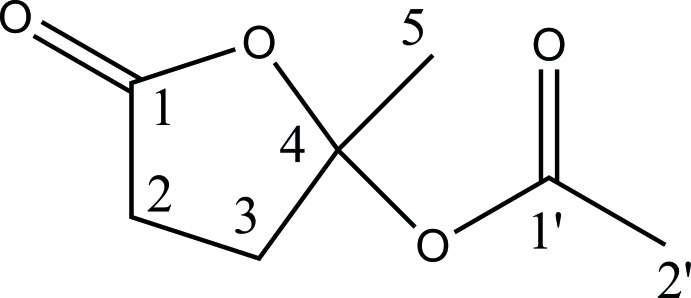


## Experimental

### 

#### Crystal data

C_7_H_10_O_4_
*M* *_r_* = 158.15
Monoclinic, 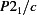

*a* = 5.86715 (15) Å
*b* = 12.7280 (3) Å
*c* = 10.2756 (3) Å
β = 106.020 (3)°
*V* = 737.55 (3) Å^3^
*Z* = 4
Cu *K*α radiation
μ = 1.00 mm^−1^
*T* = 120 K
0.19 × 0.12 × 0.07 mm

#### Data collection

Agilent SuperNova (Dual, Cu at zero, Atlas) diffractometer
Absorption correction: gaussian (*CrysAlis PRO*; Agilent, 2013 ▶) *T*_min_ = 0.812, *T*_max_ = 1.000
4959 measured reflections
1469 independent reflections
1350 reflections with *I* > 2σ(*I*)
*R*_int_ = 0.031

#### Refinement

*R*[*F* ^2^ > 2σ(*F* ^2^)] = 0.032
*wR*(*F* ^2^) = 0.084
*S* = 1.03
1469 reflections
102 parameters
H-atom parameters constrained
Δρ_max_ = 0.24 e Å^−3^
Δρ_min_ = −0.22 e Å^−3^

### 

Data collection: *CrysAlis PRO* (Agilent, 2013 ▶); cell refinement: *CrysAlis PRO*; data reduction: *CrysAlis PRO*; program(s) used to solve structure: *SHELXS97* (Sheldrick, 2008 ▶); program(s) used to refine structure: *SHELXL2012* (Sheldrick, 2008 ▶); molecular graphics: *ORTEP* in *WinGX* (Farrugia, 2012 ▶); software used to prepare material for publication: *SHELXL2012* and *PLATON* (Spek, 2009 ▶).

## Supplementary Material

Crystal structure: contains datablock(s) global, I. DOI: 10.1107/S1600536813013561/cv5410sup1.cif

Structure factors: contains datablock(s) I. DOI: 10.1107/S1600536813013561/cv5410Isup2.hkl

Supplementary material file. DOI: 10.1107/S1600536813013561/cv5410Isup3.cml

Additional supplementary materials:  crystallographic information; 3D view; checkCIF report

## Figures and Tables

**Table 1:** Hydrogen-bond geometry (Å, °)

*D*—H⋯*A*	*D*—H	H⋯*A*	*D*⋯*A*	*D*—H⋯*A*
C3—H3*B*⋯O2^i^	0.99	2.68	3.5827 (13)	152
C1—H1*B*⋯O3^ii^	0.98	2.63	3.4617 (14)	142

Symmetry codes: (i) 
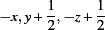
; (ii) 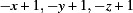.

**Fig. 1.**
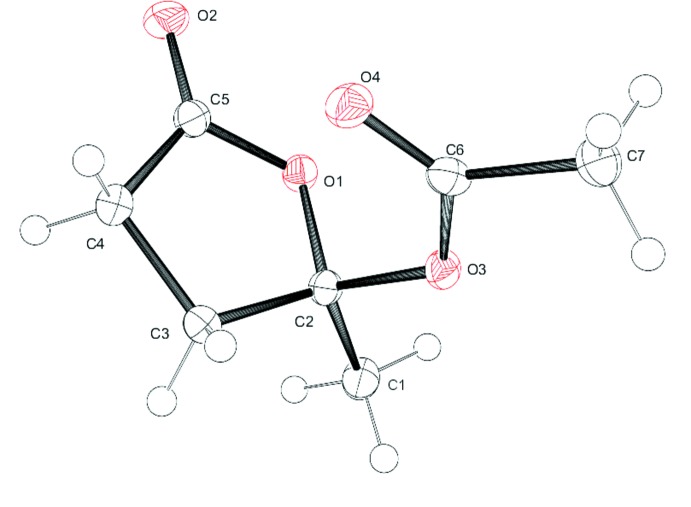
ORTEP (Farrugia, 2012) view of the title molecule showing the atomic numbering and 50% probability displacement ellipsoids.

## supplementary crystallographic information

### Comment

Acetoxy-γ-valerolactone (2-methyl-5-oxotetrahydrofuran-2-yl acetate) was first described by Bredt (1886) and became of interest during our investigation of novel, renewable levulinyl cellulose esters. Esterification of cellulose in the presence of levulinic acid and an aliphatic anhydride affords a mixed cellulose levulinyl ester which we have shown has particular utility in architectural coatings [Glenny *et al.*, 2012]. Levulinyl acetyl cellulose was generated by the sulfuric acid catalysed esterification of cellulose in the presence of acetic anhydride and levulinic acid. Analysis of this reaction mixture indicated that a valero-lactone species predominated rather than the anticipated mixture of anhydrides.

Acetoxy-γ-valerolactone was isolated by flash chromatography and identified as the major species in the reaction solution and has been assigned as the esterifying reagent. The generation of acetoxy-γ-valerolactone from acetic anhydride and levulinic acid had previously been reported (Rasmussen & Brattain, 1949) and also had been shown to be an esterifying agent forming levulinyl and acetyl amides (Suami & Day, 1959). When isolated in our hands, acetoxy-γ-valerolactone remained as a super cooled liquid, much like levulinic acid which exists as a light yellow solid or liquid, but will crystalize and displays a melting point between 30–33°C. Bell and Covington (1975) described the material as a solid with a melting point between 75–76°C but without supporting crystal structure data. The molecule was therefore crystallized from DCM and petroleum ether and the crystal structure elucidated. This confirmed the molecular structure and assisted with investigation into its esterification chemistry.

The title compound, C_7_H_10_O_4_, crystallizes with one unique molecule per asymetric unit. The five-membered ring adopts a flattened envelope conformation with O1 atom as a flap which deviates by 0.128 (1) Å from the mean plane *P* formed by the four C atoms. The acetate fragment is oriented in such a way that its mean plane and plane *P* are almost perpendicular to each other with an interplanar angle of 83.18 (7)°. There are no closely related structures in the Cambridge Structural Database, the closest being 5-(1-adamantyl)-5-ethoxytetrahydrofuran-2-one, LAGQUG (Cai *et al.*, 2004).

In the crystal, weak C_methyl_—*H*···O_acetate_ hydrogen bonds link the molecules into centrosymmetric dimers with the well known *R*^2^_2_(8) motif (Bernstein *et al.*, 1995), and weak C_methylene_—H···O_ketone_ interactions (Table 1) link further these dimers into layers parallel to (102). Table 1.

### Experimental

Levulinic acid (5.24 g, 45.1 mmol), acetic anhydride (3.46, 33.9 mmol) and concentrated sulfuric acid (12.9 mg, 129µmol) were placed in a 50 ml round bottom flask. The solution was heated to 120°C for 10 min with stirring, then quenched with 8 ml of a 5% Mg(OAc)_2_ solution in 50/50 acetic acid water. The reaction solution was extracted with DCM recovering an orange brown liquid. A portion of the recovered material was purified with flash chromatography; the column was packed with 40–63µm silica, (Davisil) to the dimensions of 110x35 mm. A gradient solvent system was used (petroleum ether/ethyl acetate 60/40 (400 ml), 50/50 (200 ml), 30/70 (200 ml), 10/90 (200 ml)) to separate and elute the 4-acetoxy-γ-valerolactone (Rf 0.42 in 60/40 petroleum ether/ethyl acetate). Suitable crystals were obtained by recrystallization from DCM and petroleum ether.

4-Acetoxy-γ-valerolactone m.p. 72–74°C (DSC): ^1^H NMR (500 MHz, CDCl_3_): δ 1.77 (s, H-3), 2.06 (s, H-1'), 2.31 (ddd, H-2, *J* 8.8, 10.6, 18.7 Hz), 2.61 (m, H-1 and H-2), 2.87 (ddd, H-1, *J* 7.75, 9.95, 17.6 Hz); ^13^C NMR δ 21.6 (C-1',OC(O)CH_3_), 26.1 (C-5,CCH_3_), 28.5 (C-2, CH_2_CH_2_), 32.7 (C-3, CH_2_C), 108.4 (C-4, Quaternary), 169.2 (C-1'), 175.4 (C-1); TOF-HRMS found 181.0472; [C_7_H_10_NaO_4_]+ calc. 181.0477.

### Refinement

All methyl H atoms were constrained to an ideal geometry (C—H = 0.98 Å) with *U*_iso_(H) = 1.5*U*_eq_(C), but were allowed to rotate freely about the adjacent C—C bond. All other C bound H atoms were placed in geometrically idealized positions and constrained to ride on their parent atoms with C—H distances of 0.99 Å and with *U*_iso_(H) = 1.2*U*_eq_(C).

### Crystal data

**Table:**

C_7_H_10_O_4_	*F*(000) = 336
*M**_r_* = 158.15	*D*_x_ = 1.424 Mg m^−^^3^
Monoclinic, *P*2_1_/*c*	Cu *K*α radiation, λ = 1.54184 Å
*a* = 5.86715 (15) Å	Cell parameters from 3083 reflections
*b* = 12.7280 (3) Å	θ = 5.7–73.5°
*c* = 10.2756 (3) Å	µ = 1.00 mm^−^^1^
β = 106.020 (3)°	*T* = 120 K
*V* = 737.55 (3) Å^3^	Block, colourless
*Z* = 4	0.19 × 0.12 × 0.07 mm

### Data collection

**Table:**

Agilent SuperNova (Dual, Cu at zero, Atlas) diffractometer	1469 independent reflections
Radiation source: SuperNova (Cu) X-ray Source	1350 reflections with *I* > 2σ(*I*)
Mirror monochromator	*R*_int_ = 0.031
Detector resolution: 5.3250 pixels mm^-1^	θ_max_ = 73.8°, θ_min_ = 5.7°
ω scans	*h* = −7→7
Absorption correction: gaussian (*CrysAlis PRO*; Agilent, 2013)	*k* = −13→15
*T*_min_ = 0.812, *T*_max_ = 1.000	*l* = −12→12
4959 measured reflections	

### Refinement

**Table:**

Refinement on *F*^2^	Primary atom site location: structure-invariant direct methods
Least-squares matrix: full	Secondary atom site location: difference Fourier map
*R*[*F*^2^ > 2σ(*F*^2^)] = 0.032	Hydrogen site location: inferred from neighbouring sites
*wR*(*F*^2^) = 0.084	H-atom parameters constrained
*S* = 1.03	*w* = 1/[σ^2^(*F*_o_^2^) + (0.0434*P*)^2^ + 0.1941*P*] where *P* = (*F*_o_^2^ + 2*F*_c_^2^)/3
1469 reflections	(Δ/σ)_max_ < 0.001
102 parameters	Δρ_max_ = 0.24 e Å^−^^3^
0 restraints	Δρ_min_ = −0.22 e Å^−^^3^

### Special details

**Table:**

Experimental. Absorption correction: CrysAlis PRO (Agilent, 2013); numerical absorption correction based on gaussian integration over a multifaceted crystal model
Geometry. All e.s.d.'s (except the e.s.d. in the dihedral angle between two l.s. planes) are estimated using the full covariance matrix. The cell e.s.d.'s are taken into account individually in the estimation of e.s.d.'s in distances, angles and torsion angles; correlations between e.s.d.'s in cell parameters are only used when they are defined by crystal symmetry. An approximate (isotropic) treatment of cell e.s.d.'s is used for estimating e.s.d.'s involving l.s. planes.

### Fractional atomic coordinates and isotropic or equivalent isotropic displacement parameters (Å^2^)

**Table:**

	*x*	*y*	*z*	*U*_iso_*/*U*_eq_	
O1	0.25087 (13)	0.23353 (6)	0.37683 (8)	0.01787 (19)	
O2	0.01399 (17)	0.10362 (7)	0.27499 (9)	0.0268 (2)	
O3	0.32805 (14)	0.40425 (6)	0.32322 (8)	0.0179 (2)	
O4	0.04509 (15)	0.35237 (6)	0.13677 (8)	0.0226 (2)	
C1	0.4004 (2)	0.36108 (9)	0.55163 (11)	0.0217 (3)	
H1A	0.3518	0.3185	0.6188	0.033*	
H1B	0.3959	0.4356	0.5747	0.033*	
H1C	0.5620	0.3418	0.5514	0.033*	
C2	0.23376 (19)	0.34162 (8)	0.41354 (11)	0.0166 (2)	
C3	−0.0297 (2)	0.36272 (9)	0.40270 (11)	0.0187 (2)	
H3A	−0.0526	0.3758	0.4932	0.022*	
H3B	−0.0882	0.4243	0.3442	0.022*	
C4	−0.1589 (2)	0.26306 (9)	0.34032 (12)	0.0206 (2)	
H4A	−0.2462	0.2319	0.4007	0.025*	
H4B	−0.2728	0.2785	0.2516	0.025*	
C5	0.0322 (2)	0.18993 (9)	0.32378 (11)	0.0187 (2)	
C6	0.2205 (2)	0.40306 (8)	0.18841 (11)	0.0185 (2)	
C7	0.3555 (2)	0.46800 (9)	0.11343 (12)	0.0236 (3)	
H7A	0.4708	0.4236	0.0862	0.035*	
H7B	0.4391	0.5245	0.1723	0.035*	
H7C	0.2451	0.4984	0.0327	0.035*	

### Atomic displacement parameters (Å^2^)

**Table:**

	*U*^11^	*U*^22^	*U*^33^	*U*^12^	*U*^13^	*U*^23^
O1	0.0182 (4)	0.0148 (4)	0.0212 (4)	0.0003 (3)	0.0065 (3)	0.0008 (3)
O2	0.0346 (5)	0.0185 (4)	0.0294 (5)	−0.0052 (3)	0.0121 (4)	−0.0058 (3)
O3	0.0204 (4)	0.0174 (4)	0.0163 (4)	−0.0029 (3)	0.0059 (3)	0.0013 (3)
O4	0.0262 (4)	0.0233 (4)	0.0172 (4)	−0.0041 (3)	0.0043 (3)	−0.0009 (3)
C1	0.0218 (6)	0.0257 (6)	0.0163 (5)	−0.0040 (4)	0.0032 (4)	0.0019 (4)
C2	0.0191 (5)	0.0141 (5)	0.0173 (5)	−0.0015 (4)	0.0061 (4)	0.0007 (4)
C3	0.0189 (5)	0.0184 (5)	0.0197 (5)	0.0003 (4)	0.0069 (4)	−0.0021 (4)
C4	0.0182 (5)	0.0211 (5)	0.0223 (5)	−0.0024 (4)	0.0054 (4)	−0.0022 (4)
C5	0.0219 (5)	0.0185 (5)	0.0163 (5)	−0.0031 (4)	0.0065 (4)	0.0011 (4)
C6	0.0229 (6)	0.0160 (5)	0.0170 (5)	0.0022 (4)	0.0064 (4)	0.0004 (4)
C7	0.0293 (6)	0.0226 (6)	0.0207 (5)	−0.0026 (5)	0.0099 (5)	0.0023 (4)

### Geometric parameters (Å, º)

**Table:**

O1—C5	1.3660 (14)	C3—C4	1.5253 (15)
O1—C2	1.4373 (13)	C3—H3A	0.9900
O2—C5	1.2000 (15)	C3—H3B	0.9900
O3—C6	1.3546 (14)	C4—C5	1.5025 (16)
O3—C2	1.4443 (13)	C4—H4A	0.9900
O4—C6	1.2063 (15)	C4—H4B	0.9900
C1—C2	1.5049 (15)	C6—C7	1.4972 (15)
C1—H1A	0.9800	C7—H7A	0.9800
C1—H1B	0.9800	C7—H7B	0.9800
C1—H1C	0.9800	C7—H7C	0.9800
C2—C3	1.5424 (15)		
			
C5—O1—C2	111.57 (8)	H3A—C3—H3B	108.8
C6—O3—C2	119.86 (8)	C5—C4—C3	105.24 (9)
C2—C1—H1A	109.5	C5—C4—H4A	110.7
C2—C1—H1B	109.5	C3—C4—H4A	110.7
H1A—C1—H1B	109.5	C5—C4—H4B	110.7
C2—C1—H1C	109.5	C3—C4—H4B	110.7
H1A—C1—H1C	109.5	H4A—C4—H4B	108.8
H1B—C1—H1C	109.5	O2—C5—O1	120.27 (11)
O1—C2—O3	107.02 (8)	O2—C5—C4	129.16 (11)
O1—C2—C1	109.32 (9)	O1—C5—C4	110.56 (9)
O3—C2—C1	104.52 (9)	O4—C6—O3	123.78 (10)
O1—C2—C3	106.79 (8)	O4—C6—C7	125.25 (10)
O3—C2—C3	114.29 (9)	O3—C6—C7	110.90 (10)
C1—C2—C3	114.62 (9)	C6—C7—H7A	109.5
C4—C3—C2	104.93 (9)	C6—C7—H7B	109.5
C4—C3—H3A	110.8	H7A—C7—H7B	109.5
C2—C3—H3A	110.8	C6—C7—H7C	109.5
C4—C3—H3B	110.8	H7A—C7—H7C	109.5
C2—C3—H3B	110.8	H7B—C7—H7C	109.5
			
C5—O1—C2—O3	−112.79 (9)	C1—C2—C3—C4	−128.27 (10)
C5—O1—C2—C1	134.56 (9)	C2—C3—C4—C5	2.08 (11)
C5—O1—C2—C3	10.02 (11)	C2—O1—C5—O2	172.31 (10)
C6—O3—C2—O1	62.87 (11)	C2—O1—C5—C4	−8.92 (12)
C6—O3—C2—C1	178.76 (9)	C3—C4—C5—O2	−177.45 (11)
C6—O3—C2—C3	−55.15 (12)	C3—C4—C5—O1	3.91 (12)
O1—C2—C3—C4	−7.04 (11)	C2—O3—C6—O4	0.50 (16)
O3—C2—C3—C4	111.11 (10)	C2—O3—C6—C7	−176.52 (9)

### Hydrogen-bond geometry (Å, º)

**Table:**

*D*—H···*A*	*D*—H	H···*A*	*D*···*A*	*D*—H···*A*
C3—H3*B*···O2^i^	0.99	2.68	3.5827 (13)	152
C1—H1*B*···O3^ii^	0.98	2.63	3.4617 (14)	142

Symmetry codes: (i) −*x*, *y*+1/2, −*z*+1/2; (ii) −*x*+1, −*y*+1, −*z*+1.
